# Sublingual Administration of Desmopressin Oral Disintegrating Tablet in a Neonate With Central Diabetes Insipidus

**DOI:** 10.7759/cureus.87902

**Published:** 2025-07-14

**Authors:** Daisuke Watanabe, Hideaki Yagasaki, Mari Tsukahara, Hiromune Narusawa, Takeshi Inukai

**Affiliations:** 1 Pediatrics, University of Yamanashi, Chuo, JPN

**Keywords:** 1-deamino-8-d-arginine vasopressin, minirin melt®, neonatal central diabetes insipidus, orally disintegrating tablet, sublingual administration

## Abstract

Precise titration of desmopressin (1-deamino-8-D-arginine vasopressin; DDAVP) is essential for managing neonatal central diabetes insipidus (CDI). Although oral administration is increasingly considered an alternative to the traditional intranasal route, the differences between DDAVP formulations and administration methods are often unclear in clinical practice. This complicates efforts to establish effective sublingual treatment protocols using oral disintegrating tablets (ODTs) in neonates. Here, we present the case of a Japanese neonate with CDI, semilobar holoprosencephaly, and a cleft lip and palate. The patient exhibited hypotonic polyuria, hypernatremia, elevated serum osmolality, and low plasma arginine vasopressin (AVP) levels within the first week of life. At 30 days of age, we dissolved a 60-µg DDAVP ODT in 5 mL of water and administered it sublingually. The initial dose of 3 µg/kg/day was titrated to 2 µg/kg/day based on the patient's clinical response. Close monitoring enabled fine adjustment of dosing. The patient achieved a stable fluid balance and did not exhibit signs of hyponatremia, seizures, or other adverse events. This case supports the potential utility of sublingual DDAVP ODTs in neonates and underscores the necessity of establishing standardized preparation and dosing protocols, which will require further clinical experience.

## Introduction

Central diabetes insipidus (CDI) is characterized by a deficiency in arginine vasopressin (AVP) resulting in polyuria due to impaired free water retention [1]. Desmopressin (1-deamino-8-D-arginine vasopressin; DDAVP), a synthetic AVP analog, remains the standard of care for CDI [2]. DDAVP is available in various formulations, including intranasal solutions, injectable solutions, oral tablets, and oral disintegrating tablets (ODTs) [3]. The available oral formulations of DDAVP come in two forms: oral solid tablets (100 or 200 μg) and ODTs (Minirin Melt® 60 or 120 μg). The oral solid tablet is swallowed, while the ODT dissolves under the tongue within seconds. Minirin Melt® is currently widely used in both pediatric and adult patients, but not in neonates.

Delivery of DDAVP to neonates is challenging because conventional routes and standard formulations are not tailored to the minute dose requirements. Traditionally, diluted intranasal DDAVP solutions (1-10 μg/dose) administered via a rhinal tube have been used for precise dose adjustment [4]. Intranasal administration results in variable absorption and inconsistent efficacy in neonates with upper respiratory tract infections, midline defects or nasal anatomical abnormalities [5].

Recently, "oral" administration has emerged as an alternative to the traditional intranasal route. However, many reports use inconsistent terminology and fail to clearly specify the formulation or route of administration. In several cases, intranasal or injectable solutions have been administered via nasogastric, buccal, or other oral routes, but are generally still referred to as "oral" DDAVP [5-8]. Additionally, the distinction between oral solid tablets and ODTs is often overlooked. These variations in terminology and delivery methods make it difficult to accurately evaluate the efficacy of sublingual DDAVP ODT administration in neonates.

One reported method involves dissolving a 60-μg DDAVP ODT in 3-5 mL of water and administering 2-5 μg/kg/day in two divided doses [4]. This method may allow for precise dose adjustment in neonates. However, even in reports describing similar dosing strategies, the exact combination of sublingual administration, ODT formulation, and neonatal patients is rarely detailed [9-11]. Sublingual DDAVP ODT administration may be an innovative approach to treating neonatal CDI, though its broader clinical applicability remains understudied. Additional well-documented cases are needed to evaluate the clinical utility of sublingual DDAVP ODT administration in neonates. Here, we present a case of a neonate with CDI who was treated with diluted DDAVP ODT administered sublingually.

## Case presentation

**Figure 1:**
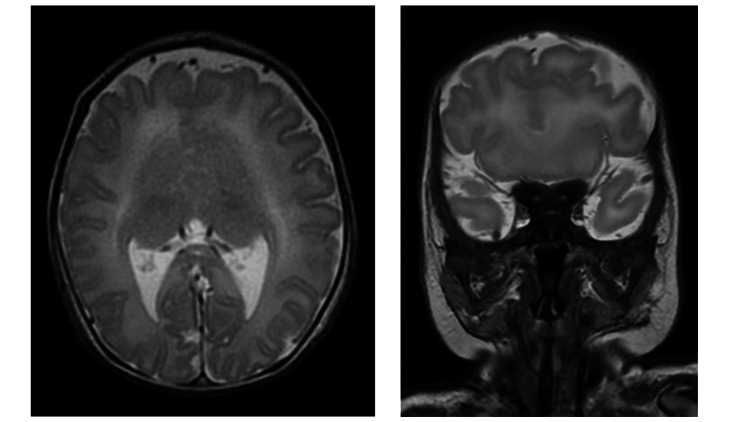
Brain MRI Axial (left) and coronal (right) images show dysplasia of the anterior commissure and fusion of the bilateral frontal lobes, findings consistent with semilobar holoprosencephaly. These findings indicate abnormalities in brain midline development, which could affect the hypothalamic-pituitary axis and help explain the cause of CDI. CDI: Central diabetes insipidus

A female infant was born at 38 weeks' gestation with a birth weight of 2,660 g. She was the third child of healthy, unrelated parents. The pregnancy was uneventful except for prenatal suspicion of agenesis of the corpus callosum. Apgar scores were 8 and 10 at one and five minutes, respectively. The infant did not require respiratory support after birth. A cleft lip and palate was noted at birth. Brain MRI revealed dysplasia of the anterior commissure and fusion of the bilateral frontal lobes, leading to the diagnosis of semilobar holoprosencephaly (Figure 1).

At approximately one week of age, the infant developed hypotonic polyuria. Laboratory data are summarized in Table 1. Serum sodium and serum osmolality were increased, whereas urine osmolality and plasma AVP levels were decreased. Other pituitary hormone levels were within normal limits. Chromosomal and microarray analyses revealed no significant abnormalities.

**Table 1:** Summary of laboratory data Elevated blood sodium and osmolality, together with low urine osmolality, were indicative of CDI. AVP: Arginine Vasopressin; TSH: Thyroid stimulating hormone; ACTH: Adrenocorticotropic hormone; IGF-1: Insulin-like growth factor 1; CDI: Central diabetes insipidus

Parameter	Day 7 Value	Reference Range
Serum Sodium (mEq/L)	151	136–147
Serum Osmolality (mOsm/kg)	305	276–292
Urine Osmolality (mOsm/kg)	90	100-400
Plasma AVP (pg/mL)	0.7	0.5-3
TSH (µIU/mL)	1.85	0.61–4.23
Free T3 (pg/mL)	4.63	2.3–5.1
Free T4 (ng/mL)	1.57	0.78–1.63
ACTH (pg/mL)	53.9	10–60
Cortisol (µg/mL)	6.5	3.6–18.1
IGF-1 (ng/mL)	75	15–154 (age-specific)

**Figure 2:**
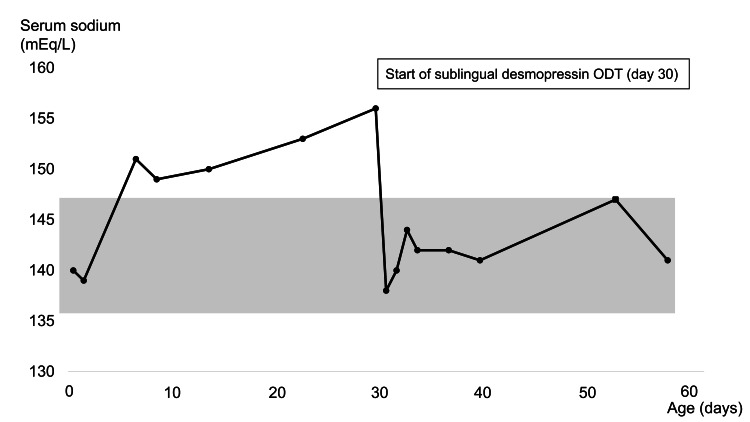
Clinical course of serum sodium levels from birth to 60 days of age The black circles connected by a solid line represent measured serum sodium concentrations (in mEq/L). The gray shaded area denotes the reference range for serum sodium. Sublingual administration of DDAVP ODTs began on day 30 of life and continued thereafter. This treatment resulted in the normalization and stable maintenance of serum sodium concentrations within the reference range. This figure demonstrates the effectiveness of sublingual DDAVP ODT in correcting hypernatremia in neonatal CDI. DDAVP: Desmopressin; ODT: Oral disintegrating tablets; CDI: Central diabetes insipidus

On day 30 of life, a 60-μg DDAVP ODT was dissolved in 5 mL of water and administered sublingually at a dose of 3 µg/kg/day in two divided doses. Intranasal administration was not used because the cleft palate prevented effective drug delivery. Serum sodium levels normalized within 24 hours of the initial treatment dose. The dose was later adjusted to 2 µg/kg/day due to fluctuations in serum sodium levels and urine output. This adjustment stabilized these parameters without adverse effects such as oliguria, hyponatremia, or seizures (Figure 2). The patient was diagnosed with CDI based on clinical and biochemical findings. Adipsic hypernatremia was considered as a differential diagnosis, but the consistent response to DDAVP was more compatible with CDI.

The patient was discharged from the hospital at two months of age. Home sublingual DDAVP ODT administration was continued without complications. At 12 months of age, she exhibits developmental delays compared to her peers. A multidisciplinary team, including pediatric endocrinologists and developmental specialists, is closely monitoring her condition.

## Discussion

We report a neonatal case of CDI treated with sublingual administration of DDAVP ODT. Our findings indicate that sublingual administration of DDAVP ODT may be a practical and reliable therapeutic option for neonatal CDI, consistent with its established efficacy in pediatric and adult populations.

The sublingual administration of DDAVP ODTs in neonates has rarely been reported. A PubMed search using the keywords "desmopressin", "neonate", and "central diabetes insipidus" from 2011 to 2025 identified 26 articles. After screening, three articles describing seven cases in neonates were included, as summarized in Table 2 [9-11]. The gestational age ranged from 28 to 40 weeks, and six of the infants had structural brain anomalies. Preparation methods varied; some studies, as in our case, dissolved a 60-µg tablet in 5 mL of water, while others divided the tablet and placed the fragments sublingually [9,10]. The variations in preparation and administration methods highlight the lack of a standardized protocol for sublingual administration in neonates. In our case, a lower-than-typical dose was sufficient to maintain stable serum sodium levels and adequate hydration, possibly due to preserved endogenous pituitary function. Despite differences in baseline conditions, dosing strategies, and serum sodium levels, all of the reported neonates achieved therapeutic effects without experiencing serious adverse events, such as hyponatremia.

**Table 2:** Comparison of sublingual administration of DDAVP ODT in neonates GA: Gestational age; BW: Birth weight; M: Male; F: Female; NA: Not available; TSH: Thyroid stimulating hormone; DDAVP: Desmopressin; ODT: Oral disintegrating tablet

Patient No.	Our patient	1 (De Waele et al. [9])	2 (De Waele et al. [9])	3 (Korkmaz et al. [10])	4 (Korkmaz et al. [10])	5 (Korkmaz et al. [10])	6 (Korkmaz et al. [10])	7 (Hanta et al. [11])
GA (week)	38	28	40	40	38	36	40	28
BW (g)	2,660	1,220	2,700	3,650	3,070	3,280	3,420	1,100
Sex	F	M	F	M	M	F	M	F
Brain abnormalities	Semilobar holoprosencephaly, Dysplasia of the anterior commissure	Meningitis/Ventriculitis, Absence of bright spot	Bilateral subdural hygroma, Schizencephaly, Polymicrogyria, Absence of bright spot	Schizencephaly, Ectopic posterior pituitary gland	Absence of the septum pellucidum	Septo-optic dysplasia, Pituitary hypoplasia	Schizencephaly, Corpus callosum agenesis	None
Midline defects or Nasal abnormalities	Cleft lip and palate	None	None	None	None	None	None	None
Abnormal other hormones	None	NA	NA	TSH	None	None	TSH	None
Serum osmolality (mOsm/kg)	305	320	322	327	320	295	296	362
Urine osmolality (mOsm/kg)	68	60	100	178	114	225	200	165
Maximum Serum Na (mEq/L)	156	164	168	178	172	160	161	170
Adverse event (Hyponatremia)	No	No	No	No	No	No	No	No
Dissolved	Water 5 mL	None	None	Water 5 mL	Water 5 mL	Water 5 mL	Water 5 mL	NA
Initial dose (μg/kg/day)	3	(60 μg/day)	(15 μg/day)	5	5	5	5	3-4
Maintenance dose (μg/kg/day)	2	(60 μg/day)	(45 μg/day)	4	3	3	3	1-2

The safety profile of sublingual DDAVP ODT administration is favorable. "Oral" DDAVP formulations have been found to be associated with a lower incidence of hyponatremia than intranasal formulations [12]. A review of 172 cases of hyponatremia-related symptoms (e.g., headache, nausea, and vomiting) associated with intranasal DDAVP was published, whereas only six cases were reported with oral formulations [13]. Oral administration results in a slower time to peak plasma concentration and a shorter duration of action than intranasal administration, which may reduce water retention and sodium imbalance [13]. Due to its lower incidence of adverse events and pharmacological advantages, oral DDAVP administration, particularly sublingual DDAVP ODT, would be a safe treatment option for CDI.

The pharmacologic characteristics of neonates differ from those of adults. In neonates, hepatic and gastrointestinal functions are both immature. Reduced enzyme activity and variable gastric pH and motility result in less predictable absorption [14-16]. Sublingual ODTs bypass the gastrointestinal tract and are less affected by hepatic and gastrointestinal functions [12,17]. Therefore, sublingual DDAVP could be a rational therapy for neonates based on their pharmacologic characteristics.

Establishing sublingual drug administration in neonates could provide a new therapeutic option in neonatal care. Though reports on sublingual drug administration are limited, some studies suggest that it is an effective treatment for neonatal opioid withdrawal syndrome [18]. Additionally, developing dedicated sublingual formulations could promote the widespread adoption of this administration method. To maximize the benefits of sublingual drug delivery, multicenter studies and standardized protocols are necessary to generate robust data on sublingual DDAVP ODT administration in neonates.

It is difficult to differentiate CDI from adipsic hypernatremia in neonates due to their overlapping clinical features, the inability to assess thirst, and the limitations of water deprivation testing [19]. Adipsic hypernatremia results from hypothalamic damage that impairs thirst perception and AVP secretion. This condition can closely resemble CDI, especially during the first few months of life [20]. In the present case, the consistent response to DDAVP and continued need for treatment were more indicative of CDI. However, diagnostic uncertainty remains due to overlapping biochemical findings. Therefore, both conditions should be considered when evaluating hypernatremia in neonates.

## Conclusions

We present a case of neonatal CDI that was treated with sublingual administration of DDAVP ODT. Our experience shows that individualized sublingual treatment with DDAVP ODT can stabilize fluid and electrolyte balance without causing adverse events, even in neonates with complex conditions. Our report contributes to clearer treatment terminology and supports improved clinical decision-making for neonatal patients by providing detailed information on the formulation, dosage, and clinical response of DDAVP ODT. Additionally, this case highlights the practical advantages of sublingual therapy when intranasal or intravenous administration is difficult or unreliable. Further clinical experience and multicenter studies are needed to establish standard neonatal dosing protocols and explore the broader applicability of this administration route in neonatal care.

## References

[1] Almutlaq N, Eugster EA (2021). Variability in oral desmopressin dose requirements in children with central diabetes insipidus. J Pediatr.

[2] Gasthuys E, Dossche L, Michelet R (2020). Pediatric pharmacology of desmopressin in children with enuresis: a comprehensive review. Paediatr Drugs.

[3] Vande Walle J, Stockner M, Raes A, Nørgaard JP (2007). Desmopressin 30 years in clinical use: a safety review. Curr Drug Saf.

[4] Di Iorgi N, Morana G, Napoli F, Allegri AE, Rossi A, Maghnie M (2015). Management of diabetes insipidus and adipsia in the child. Best Pract Res Clin Endocrinol Metab.

[5] Smego AR, Backeljauw P, Gutmark-Little I (2016). Buccally administered intranasal desmopressin acetate for the treatment of neurogenic diabetes insipidus in infancy. J Clin Endocrinol Metab.

[6] Mavinkurve M, McGrath N, Johnston N, Moloney S, Murphy NP, Hawkes CP (2017). Oral administration of diluted nasal desmopressin in managing neonatal central diabetes insipidus. J Pediatr Endocrinol Metab.

[7] Lim WY, Riba-Wolman R (2020). Intravenous formulation of desmopressin delivered via oral and g tube routes for the treatment of central diabetes insipidus: first experience in infants. Clin Endocrinol (Oxf).

[8] Korkmaz HA, Arya VB, Gönüllü A, Coşkunol F, Ozkan B (2023). Management of central diabetes insipidus in disabled children with diluted oral desmopressin lyophilisate formulation administered through nasogastric tube: a retrospective case series. Paediatr Drugs.

[9] De Waele K, Cools M, De Guchtenaere A (2014). Desmopressin lyophilisate for the treatment of central diabetes insipidus: first experience in very young infants. Int J Endocrinol Metab.

[10] Korkmaz HA, Demir K, Kılıç FK, Terek D, Arslanoğlu S, Dizdarer C, Ozkan B (2014). Management of central diabetes insipidus with oral desmopressin lyophilisate in infants. J Pediatr Endocrinol Metab.

[11] Hanta D, Törer B, Temiz F, Kılıçdağ H, Gökçe M, Erdoğan O (2015). Idiopathic central diabetes insipidus presenting in a very low birth weight infant successfully managed with lyophilized sublingual desmopressin. Turk J Pediatr.

[12] De Guchtenaere A, Van Herzeele C, Raes A, Dehoorne J, Hoebeke P, Van Laecke E, Vande Walle J (2011). Oral lyophylizate formulation of desmopressin: superior pharmacodynamics compared to tablet due to low food interaction. J Urol.

[13] Robson WL, Leung AK, Norgaard JP (2007). The comparative safety of oral versus intranasal desmopressin for the treatment of children with nocturnal enuresis. J Urol.

[14] Khan AB, Kingsley T, Caroline P (2017). Sublingual tablets and the benefits of the sublingual route of administration. J Pharm Res.

[15] Osterberg O, Savic RM, Karlsson MO, Simonsson US, Nørgaard JP, Walle JV, Agersø H (2006). Pharmacokinetics of desmopressin administrated as an oral lyophilisate dosage form in children with primary nocturnal enuresis and healthy adults. J Clin Pharmacol.

[16] Johnson TN, Bonner JJ, Tucker GT, Turner DB, Jamei M (2018). Development and applications of a physiologically-based model of paediatric oral drug absorption. Eur J Pharm Sci.

[17] Park JY, Kim KA, Park PW, Ha JM (2006). Effect of high-dose aspirin on CYP2E1 activity in healthy subjects measured using chlorzoxazone as a probe. J Clin Pharmacol.

[18] Yen E, Davis JM (2018). Finding the right treatment for neonatal abstinence syndrome: is this the right time?. J Perinatol.

[19] Djermane A, Elmaleh M, Simon D, Poidvin A, Carel JC, Léger J (2016). Central diabetes insipidus in infancy with or without hypothalamic Adipsic hypernatremia syndrome: early identification and outcome. J Clin Endocrinol Metab.

[20] Yang T, Wu W, Liu X (2024). Clinical characteristics of adipsic diabetes insipidus. Endocr Pract.

